# *In Vivo* Toxicological Analysis of the ZnFe_2_O_4_@poly(*t*BGE-*alt*-PA) Nanocomposite: A Study on Fruit Fly

**DOI:** 10.1021/acsomega.3c07111

**Published:** 2024-01-16

**Authors:** Shaily Chauhan, Seekha Naik, Rohit Kumar, Janne Ruokolainen, Kavindra Kumar Kesari, Monalisa Mishra, Piyush Kumar Gupta

**Affiliations:** † Department of Life Sciences, Sharda School of Basic Sciences and Research, Sharda University, Greater Noida 201310, Uttar Pradesh , India; ‡ Centre for Development of Biomaterials, Sharda University, Greater Noida 201310, Uttar Pradesh , India; § Department of Life Science, National Institute of Technology, Rourkela 769008, Odisha , India; ∥ Department of Applied Physics, School of Science, Aalto University, Espoo 02150, Finland; ⊥ Research and Development Cell, Lovely Professional University, Phagwara 144411, Punjab , India; # Department of Biotechnology, Graphic Era (Deemed to Be University), Dehradun 248002, Uttarakhand, India

## Abstract

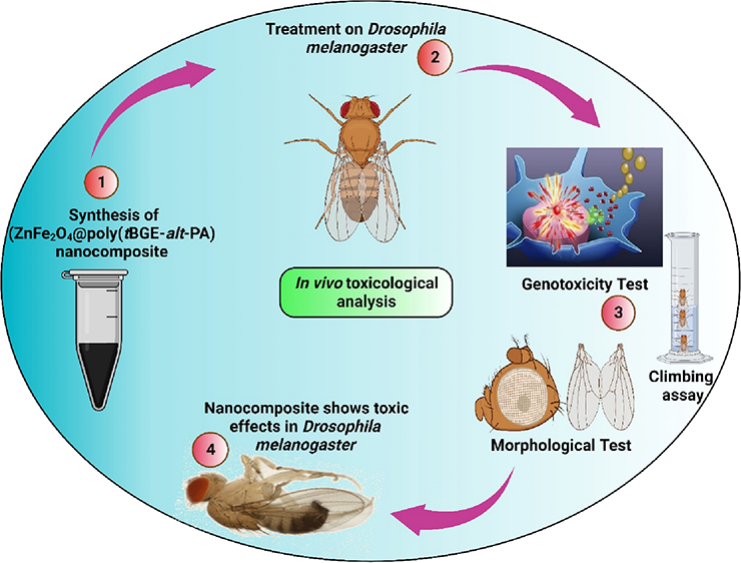

Recently, the use of hybrid nanomaterials (NMs)/nanocomposites has widely increased for the health, energy, and environment sectors due to their improved physicochemical properties and reduced aggregation behavior. However, prior to their use in such sectors, it is mandatory to study their toxicological behavior in detail. In the present study, a ZnFe_2_O_4_@poly(*t*BGE-*alt*-PA) nanocomposite is tested to study its toxicological effects on a fruit fly model. This nanocomposite was synthesized earlier by our group and physicochemically characterized using different techniques. In this study, various neurological, developmental, genotoxic, and morphological tests were carried out to investigate the toxic effects of nanocomposite on *Drosophila melanogaster*. As a result, an abnormal crawling speed of third instar larvae and a change in the climbing behavior of treated flies were observed, suggesting a neurological disorder in the fruit flies. DAPI and DCFH-DA dyes analyzed the abnormalities in the *larva’s* gut of fruit flies. Furthermore, the deformities were also seen in the wings and eyes of the treated flies. These obtained results suggested that the ZnFe_2_O_4_@poly(*t*BGE-*alt*-PA) nanocomposite is toxic to fruit flies. Moreover, this is essential to analyze the toxicity of this hybrid NM again in a rodent model in the future.

## 1 Introduction

Today, the study of the potentially harmful effects of engineered nanomaterials (NMs)/nanoparticles (NPs) on living organisms is of utmost requirement in the field of nanotoxicology.^1^ The rapid use of nanoproducts for daily purpose has increased enormously, which provide considerable concern regarding their safety in humans.^2^ Due to their small size and unique features, NMs have been used in drug delivery, tissue engineering, sensing, electronics, environmental remediation, and energy sectors.^2^ Furthermore, the toxic behavior of NMs within the healthy cells, tissues, and organs may result from their reduced size, morphology, physicochemical properties, and functionalization with exterior functional groups. Commonly, chemically synthesized NMs can cause more severe damage to human cells in contrast to biologically synthesized NMs.^3^

Metal (M)/metal oxide (MO) NPs can be synthesized by chemical as well as biological methods using different metal salts.^4^ A few examples of such NPs are ZnFe_2_O_4_, MnFe_2_O_4_,^5^ ZnO, Fe_3_O_4_, Fe_2_O_4_, CuO, ZnO, Ag, Cu, and Au.^6,7^ These NPs possess surface plasmon resonance properties and are flexible in surface functionalization. Furthermore, their biocompatible nature leads to their broader use as anticancer, antibacterial,^8^ antiviral, antifungal, anti-insecticidal, antiprotozoans, or antileishmanial activities.^9−11^ Moreover, the use of M/MO NPs has been limited due to their slower degradation and aggregation properties. Therefore, to overcome such limitations, M/MO NPs were coated with polymers to develop nanocomposite for enhancing their physicochemical properties.^5,12^ Due to this, these hybrid NMs can be utilized broadly for healthcare and environmental applications further.

Generally, metallopolymer composites possess the combined properties of both polymers such as flexibility, solubility, biodegradability, and biocompatibility and metal NPs like thermostability, electrical, optical, and catalytic properties.^13−15^ They have been utilized for biosensing, bioimaging, bioelectronics, drug delivery, tissue engineering,^16^ and bioremediation applications.^9−11^ The polymer components of the metallopolymer nanocomposite are amphiphilic, biodegradable, and cytocompatible in nature. Among different polymers, polyesters have been used majorly to fabricate metallopolymer nanocomposites.^17−19^ Examples of such polyesters are poly(ε-caprolactone), poly(lactic-*co*-glycolic acid), poly(lactic acid), and poly(glycolic acid).^20,21^ In the past few years, several other polyesters have been also synthesized using a metal-free copolymerization approach, i.e., cost-effective, eco-friendly, and fast.^17,22^ By this reaction, the obtained polyesters are free from metal contamination and have been utilized for drug delivery applications.^17−19^ Next, the metal components of the metallopolymer nanocomposite are biocompatible, hemocompatible, and catalytic in nature. In such nanocomposites, MO NPs have contributed largely in the past few years.^23^

In an earlier study, a ZnFe_2_O_4_@poly(*t*BGE-*alt*-PA) composite, i.e., a metallopolyester nanocomposite, was physically synthesized by our group where poly(*t*BGE-*alt*-PA) copolymer is a metal-free semiaromatic polyester and ZnFe_2_O_4_ are MO NPs.^12^ The obtained hybrid NM was characterized by different physicochemical techniques by which its thermostability and chemical interactions between both components, phase transition, and magnetization were studied. Furthermore, biological characterization was also carried out, which showed the biocompatible and hemocompatible nature of nanocomposite.^12^ However, an in-depth toxicological analysis of this nanocomposite is required for an *in vivo* model. Keeping this in view, in the present study, various tests will be performed to study the toxicological behavior of the ZnFe_2_O_4_@poly(*t*BGE-*alt*-PA) composite in a fruit fly model, *viz*., *Drosophila melanogaster*.^24^ These tests will be carried out on genetic, cellular, morphological, and developmental levels as the fruit fly is a suitable model for studying NM/NP toxicity.^25−27^ Based on the results, the nanocomposite will be tested further in a rodent model soon.

## 2 Experimental Section

### 2.1 Synthesis and Characterizations of the ZnFe_2_O_4_@poly(*t*BGE-*alt*-PA) Nanocomposite

The ZnFe_2_O_4_@poly(*t*BGE-*alt*-PA) nanocomposite was synthesized earlier by our group using the physical method and further characterized physicochemically by different techniques.^12^ For instance, Fourier transform infrared spectroscopy (FTIR) was used to study the preparation of nanocomposite. X-ray diffraction (XRD) was applied to analyze the crystalline nature of nanocomposite. Differential scanning colorimetry (DSC) was performed to analyze the chemical interactions between ZnFe_2_O_4_ NPs and poly(*t*BGE-*alt*-PA) copolymers. A thermogravimetric analyzer (TGA) was used to study the thermostability of nanocomposite.^12^ Furthermore, the biocompatibility and hemocompatibility of nanocomposite were studied.^12^

### 2.2 *In Vivo* Studies on Fruit Fly

#### 2.2.1 *Drosophila* Strains and Culture Conditions

For conducting the experiments on *D. melanogaster*, the *Oregon R* fly strain was used. The sterile fly food is made up of corn (5 g), sucrose (4 g), yeast (2.5 g), and agar (0.8 g) in 100 mL of distilled water to grow both larval and adult flies.^28,29^ In addition, nepagine and propionic acid were also mixed in the food to prevent bacterial and fungal contamination. The male and female flies in a 5:7 ratio were taken in the food vials. All flies were kept in an environment with a temperature of 24 °C, 60% relative humidity, and a 12 h light/dark cycle.^30^

#### 2.2.2 Treatment of Flies with a ZnFe_2_O_4_@poly(*t*BGE-*alt*-PA) Composite

The toxicological behavior of a ZnFe_2_O_4_@poly(*t*BGE-*alt*-PA) nanocomposite was tested on an *Oregon R* fruit fly strain. The nanocomposite was suspended in Milli-Q water to develop a stock solution of 2 mM concentration, which was kept at 4 °C later. The experiment was conducted in four separate groups where flies of three groups were treated with different concentrations of nanocomposite mixed in food vials such as 50, 100, and 200 μM. One group of flies was not treated with nanocomposite and was considered as a control group. After treatment, every day, eggs, larvae, pupae, and adult flies were observed and their developmental cycle was noticed.^27,31^

#### 2.2.3 Larval Crawling Behavior Analysis

A larval crawling assay was performed to study the abnormalities in neurons of third instar larvae of fruit flies. The agar plates (2%) were used to analyze the path of treated larvae showing the toxicological effects of nanocomposite. Usually, the larvae move in a rhythmic crawling pattern along a straight line at a certain speed. Any change in the crawling patterns indicates neurological abnormalities.^32,33^ In this experiment, third instar larvae (*n* = 6) of each treated group (50, 100, and 200 μM) along with the control group were used during the assay. The larvae were placed in the middle of the agar plate and allowed to move one by one. To measure the crawling path, a graph paper was placed below the agar plate and observed for 1 min under the camera (Canon EOS 3000D, Japan). Then, the routes were marked with a marker, and the crawling speed was calculated per minute and represented on a histogram.^32,33^

#### 2.2.4 Larval Gut Staining for Cytotoxicity Analysis

The gut of third instar larvae was stained using 4′,6-diamidino-2-phenylindole (DAPI) (D9542, Sigma-Aldrich, Germany) and dichloro-dihydro-fluorescein diacetate (DCFH-DA) (D6883, Sigma-Aldrich, Germany) dyes to study the production of reactive oxygen species (ROS), which triggers the cell death. The larvae from each treated group along with the control group were collected and rinsed with 1× phosphate-buffered saline (PBS) to eliminate the food residue.^25,34^ The larvae’s gut was dissected on a glass slide using fine forceps under a stereomicroscope (ACCU-SCOPE Inc., Commack, New York). The dissected guts were transferred to a 1.5 mL tube containing 4% PFA (paraformaldehyde) and further kept at 4 °C overnight. The fixed guts were rinsed with 1× PBS three times for 5 min and again with PBST solution (1× PBS with 0.2% Tween 20) for 15 min. For each experiment, 10 μL of freshly prepared staining solutions like DAPI and DCFH-DA was added to the respective tube. The stained gut was kept in the dark for 5 min with DAPI and 30 min with DCFH-DA dye. Later, the stained gut was mounted on slides with glycerol and a coverslip. Finally, the slides were observed under the Stellaris confocal microscope (Leica Microsystems, Germany).^26^

#### 2.2.5 Comet Assay

Comet assay is used to study DNA damage in cells. This method analyzes the extent of DNA damage after oral ingestion of the ZnFe_2_O_4_@poly(*t*BGE-*alt*-PA) nanocomposite. In brief, third instar larvae (*n* = 30) were treated with different concentrations of nanocomposite. All larvae were collected from the treated group, and then a fine needle was used to puncture their cuticle. Next, the hemolymph was collected and kept on ice to stop melanization. The obtained hemolymph was mixed with 0.65% low melting agar by pipetting and spread over the slide for drying for 24 h.^33,35^ The slides were dipped in cold lysis buffer for 1 h in the dark to prevent further damage. Then, slides were kept in electrophoretic solution for 15 min, and later the electrophoresis was carried out for 25 min at 25 V and 300 mA. After electrophoresis, slides were washed with cold neutralizing solution (0.4 m Tris buffer pH 7.5) for 5 min and further stained with EtBr solution. At the end, the slides were visualized under the fluorescence microscope (OLYMPUS DP72, Japan) to observe the damaged DNA.^36^

#### 2.2.6 Climbing Assay

In this assay, adult flies were used to study their locomotor abnormalities and negative geotaxis behavior. In brief, adult flies (*n* = 22) of an equal number of males and females were taken from food vials treated with different concentrations of nanocomposite. These flies were transferred into a 100 mL measuring cylinder, and its mouth was sealed with a cotton plug. Next, the cylinder was tapped three times to force the flies to reach the bottom of the cylinder once they adjusted to their surroundings.^26^ The climbing pattern of flies was recorded for 10 s using a camera (Canon EOS 3000D, Japan), and then the percentages of flies that crossed the 80 mL mark in the cylinder were counted.^33,35^

#### 2.2.7 Adult Weight Analysis

The body weight of adult flies was measured, i.e., collected from different groups and compared with the control. In brief, male and female flies in equal numbers were taken from each food vial. The fly’s weight was calculated using a weighing balance, and then a graph was plotted.^28,29^

#### 2.2.8 Morphological Analysis

The adult fly from each food vial was examined under a stereomicroscope (ACCU-SCOPE Inc., Commack, New York) after developing from eclosion of pupae. The images of various body parts like the head, eye, thorax, and wings were captured to visualize the morphological abnormalities.^28,29^

### 2.3 Statistical Analysis

All the experiments were conducted three independent times. All graphs were plotted using GraphPad Prism 9.0 software (GraphPad Prism, USA). The statistical analysis between the control and treated groups was carried out by an unpaired two-tailed Student’s *t* test. The value of *p < 0.05 was considered significant. **p < 0.01, ***p < 0.001, ****p < 0.0001.

## 3 Results

### 3.1 Synthesis and Characterizations of the ZnFe_2_O_4_@poly(*t*BGE-*alt*-PA) Nanocomposite

The ZnFe_2_O_4_@poly(*t*BGE-*alt*-PA) nanocomposite was synthesized earlier by our group using the blending method and physicochemically characterized further. A FTIR study showed the synthesis of nanocomposite.^12^ XRD exhibited the crystalline nature of the composite. DSC study presented no chemical interactions between ZnFe_2_O_4_ NPs and poly(*t*BGE-*alt*-PA) copolymers in the nanocomposite. TGA study exhibited the thermostable nature of composite. Also, the composite was found to be nontoxic to healthy blood cells.^12^

### 3.2 Crawling Analysis

**Figure 1:**
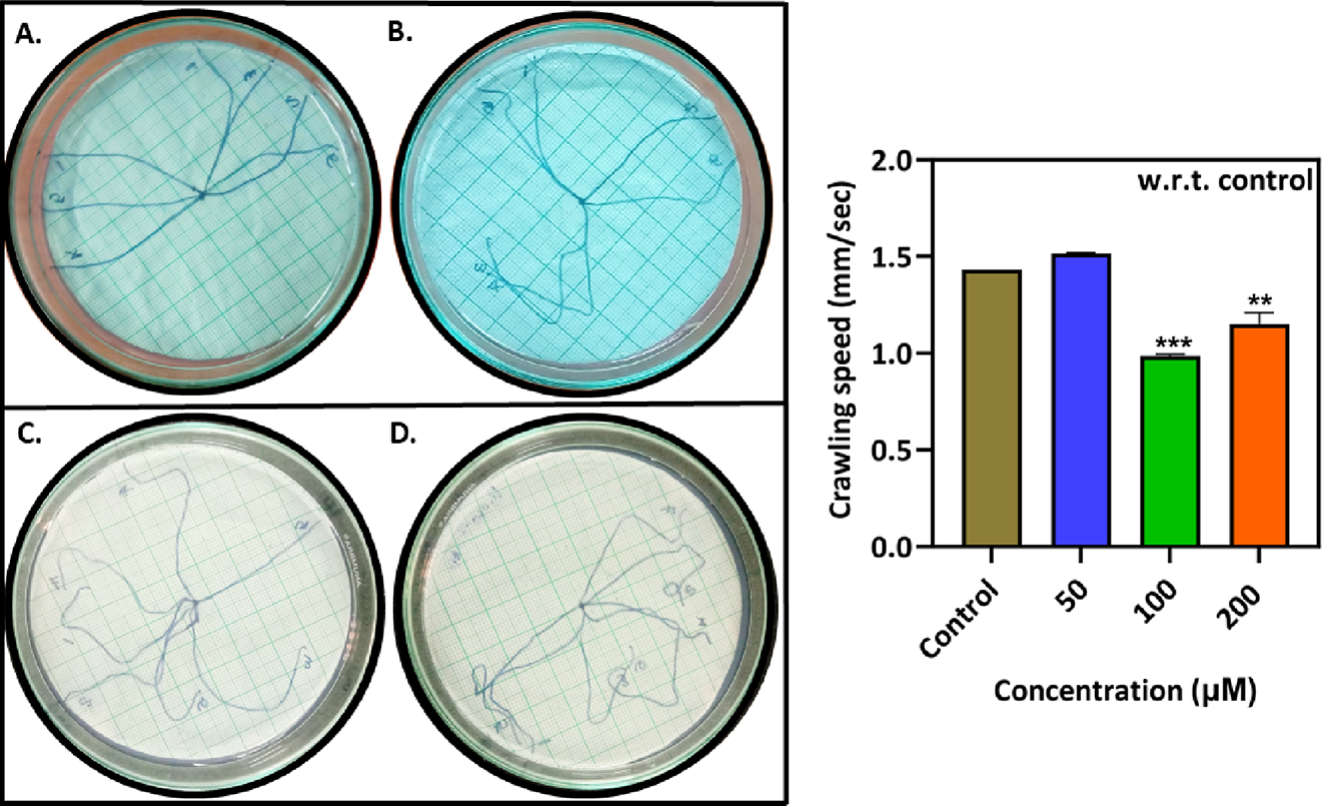
Crawling assay for third instar larvae of *Drosophila melanogaster* treated with different concentrations (B 50 μM, C 100 μM, and D 200 μM) of the ZnFe_2_O_4_@poly(*t*BGE-*alt*-PA) nanocomposite along with negative control (A). The histogram plot depicts the larval crawling speed for all of the treated and control groups.

Generally, third instar larvae of fruit flies are voracious eaters; thus, they also consume nanocomposite present in different concentrations in the given food vials. The larvae move by contracting their bodies which allows them to migrate from one to another spot. The motor neurons in the larval brain are responsible for controlling such contractions. If there is any defect in such motor neurons, then it can result in a defective crawling pattern. In this assay, the larvae of the control group move in a straight line only with minor twists, which is also seen for the larvae collected from the 50 μM group. The distance traveled by larvae collected from control and 50, 100, and 200 μM groups were measured to be 1.436 ± 0.45, 1.520 ± 0.00, 0.996 ± 0.00, and 1.220 ± 0.22 mm/s, respectively. Due to nanocomposite treatment, the larval speed was affected and more chaos was observed in the larval track pattern for the 100 and 200 μM groups compared with the control group.^32,33^ Also, several turns and stops were seen during larval movement when the nanocomposite’s concentration was increased. Furthermore, such observations indicated an alternation in the activity of motor neurons (Figure 1).

### 3.3 Larval Gut Staining

**Figure 2:**
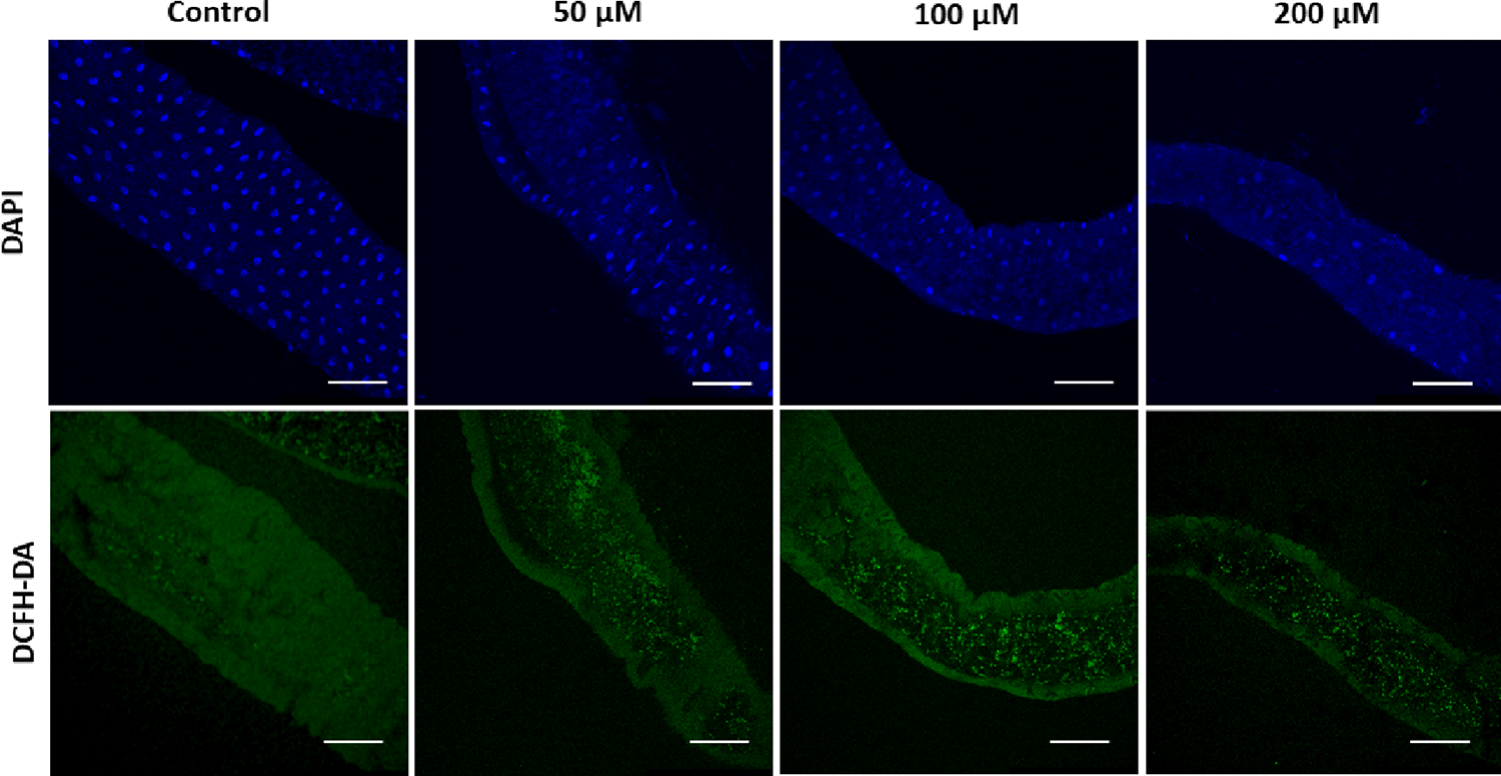
DAPI and DCFH-DA staining of the larval gut treated with different concentrations (50, 100, and 200 μM) of the ZnFe_2_O_4_@poly(*t*BGE-*alt*-PA) nanocomposite along with negative control.

DAPI binds with the A–T-rich region of DNA and provides blue color fluorescence. It stains nuclei of dead cells, thereby it can identify micronuclei and help in their countings between live and dead cells.^26^ In this experiment, DAPI staining was used to examine the nuclei of gut cells of the third instar larvae. The stained guts were visualized using a microscope that showed the significant number of nuclear damage in treated groups compared to the control group (Figure 2). Also, micronuclei were found in clusters throughout the digestive tract. Further ROS quantity was measured using the DCFH-DA dye.^25,34^ The dye concentration signifies the extent of stress, as shown in Figure 2. Based on the obtained results, the ZnFe_2_O_4_@poly(*t*BGE-*alt*-PA) nanocomposite is toxic and affects the larval cells. However, various toxicological experiments need to be carried out on other model organisms.

### 3.4 Comet Assay

DNA damage was assessed by using the comet test. The test showed that the ZnFe_2_O_4_@poly(*t*BGE-*alt*-PA) nanocomposite induced minor damage even at high doses when administered orally through food (Figure S1). These observations were seen after 24 h of treatment.^33,35^

### 3.5 Climbing Assay

**Figure 3:**
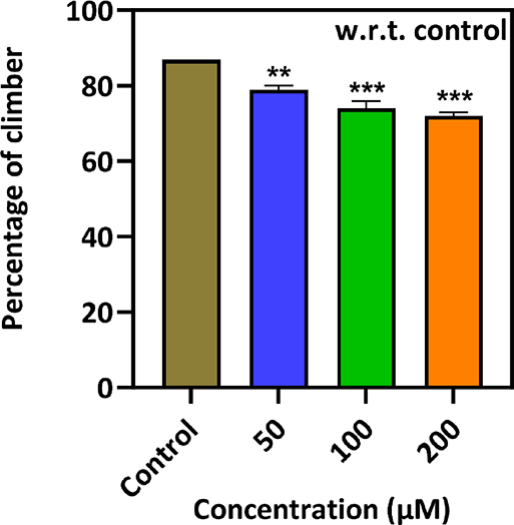
Climbing assay carried out for adult fruit flies treated with different concentrations (50, 100, and 200 μM) of the ZnFe_2_O_4_@poly(*t*BGE-*alt*-PA) nanocomposite along with control.

The neurological defects and incapability to overcome gravity in fruit flies can be studied using climbing assays. It was observed that 86.83% of flies in the control group climbed the 10 cm mark. In different treated groups (50, 100, and 200 μM), 78.96, 74.10, and 72.07% of adult flies could climb up to the 10 cm mark, respectively. It was also observed that flies of the control group can cross 16 cm in 10 s.^33,35^ Furthermore, the increase in the concentration of the nanocomposite decreased the climbing behavior of treated flies (Figure 3).

### 3.6 Adult Weight Analysis

**Figure 4:**
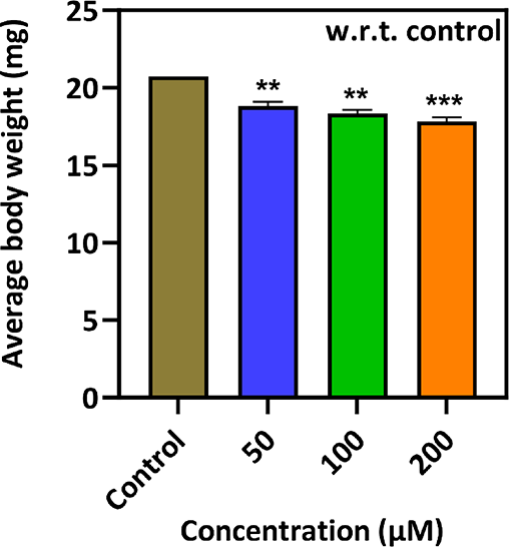
Average body weight analysis of adult fruit flies from both the control and each treated group (50, 100, and 200 μM concentrations of the ZnFe_2_O_4_@poly(*t*BGE-*alt*-PA) nanocomposite).

To analyze the body growth and size of adult flies, the body weight of flies was measured. During measurement from both the control and treated groups, 25 males and 25 females were considered. The body weight of flies in the control and 50, 100, and 200 μM treated groups were calculated to be 20.73, 18.82, 18.35, and 17.81 mg, respectively. In addition, a significant difference was seen in the body weight of the control and treated groups.^28,29^ Based on the results, an increase in the concentration of nanocomposite decreases the body weight of the flies (Figure 4).

### 3.7 Morphological Analysis

**Figure 5:**
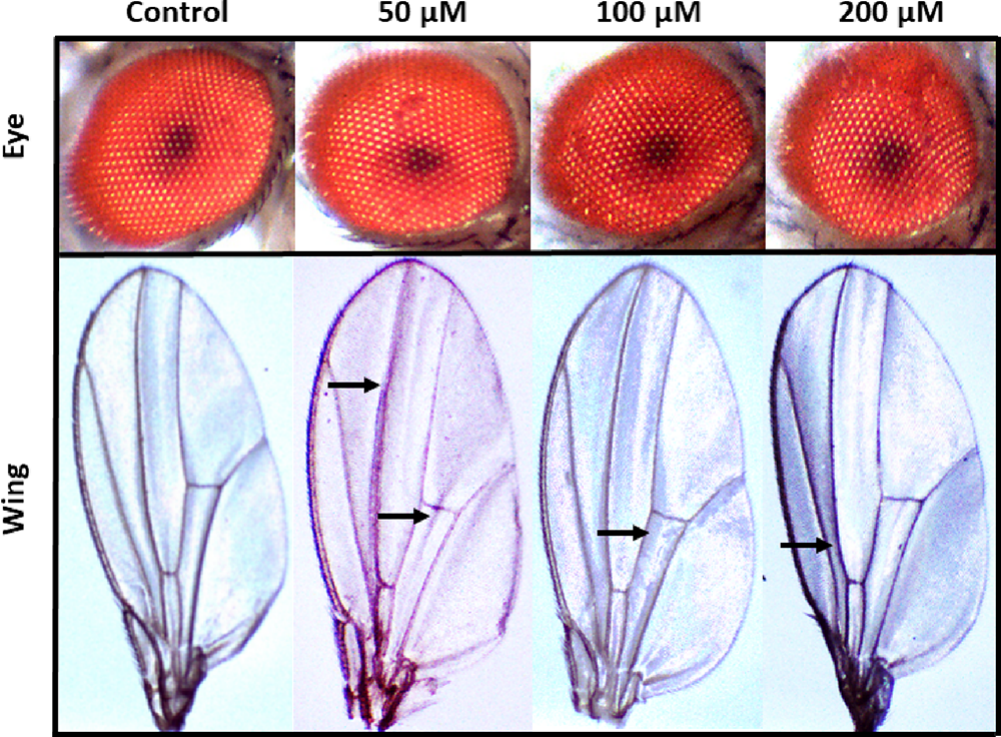
Eye coloration and wing venation patterns of *Drosophila melanogaster* studied after the treatment with different concentrations (50, 100, and 200 μM) of the ZnFe_2_O_4_@poly(*t*BGE-*alt*-PA) nanocomposite along with negative control. The arrow marks indicate the change in the wing’s venation pattern.

After nanocomposite treatment, the phenotypic pattern of fruit flies was observed in all treated and control groups.^24,37^ The venation patterns of the wings and the eye color were observed during the analysis.^28,29^ Maximum changes in the wing’s venation pattern and more eye defects were seen in the fruit flies of the 100 and 200 μM treated groups as compared to the control (Figure 5). However, small defects were seen in the fruit flies of the 50 μM treated groups (Figure 5).

## 4 Discussion

In the twenty-first century, NMs have gained a lot of attention in different fields due to their tiny size and excellent physicochemical properties like chemical composition, solubility, surface charge, and shape.^38^ Moreover, prior to using them for biomedical applications, there is a need for advanced studies to understand their toxicological behavior on human health at subcellular, cellular, tissue, and organ levels.^39^ To study the toxicological properties of NMs, several *in vitro, in vivo,* and *ex vivo* models have been developed. Among these models, the *in vivo* system played a significant role in inferring the toxicological nature of NMs at the whole-body level.^40^ However, *in vivo* models are not easy to handle and require a lot of cost to conduct the experiments and maintenance of animals. Therefore, an *in vivo* study on fruit flies like *D. melanogaster* provides a cost-effective, easy, and fast method. Due to this, *Drosophila* could be employed as a model organism in nanotoxicological research.^41^

In our study, the enhanced physicochemical features of an earlier synthesized ZnFe_2_O_4_@poly(*t*BGE-*alt*-PA) nanocomposite by our group may suggest its significant role in biomedical applications.^12,42−45^ Moreover, prior to their use, the nanocomposite was tested on adult fruit flies and their larvae to study its genotoxic, neurotoxic, and cytotoxic effects. Similar research was also conducted for the MnFe_2_O_4_@poly(*t*BGE-*alt*-PA) nanocomposite by our group previously.^26^ Like in our previous study, the ZnFe_2_O_4_@poly(*t*BGE-*alt*-PA) nanocomposite was mixed in the food vials at different concentrations. When third instar larvae were fed on nanocomposite-treated food, several changes were observed in their behaviors. As larvae had the stress of surviving, they could not transform to the adult stage due to the altered process of development.^46,47^ Furthermore, the irregular crawling pattern of third instar larvae showed the influence of nanocomposite on the mechanosensory neurons.^48^ Usually, larvae travel the straight path but the treated larvae showed a zigzag path and occasionally moved slowly as compared to nontreated larvae. Also, a reduction in the larvae’s crawling speed was seen when the concentration of nanocomposites increased in the food vials. Next, gut toxicity was observed using DCFH-DA and DAPI dyes.^49^ Several clusters of micronuclei were observed in the gut cells of treated larvae due to nuclear fragmentation. A large number of micronuclei indicates that the ZnFe_2_O_4_@poly(*t*BGE-*alt*-PA) nanocomposite is toxic in nature. However, in our previous report, no such observations were seen for the MnFe_2_O_4_@poly(*t*BGE-*alt*-PA) nanocomposite.^26^ Next, DCFH-DA dye evaluated the production of ROS within the gut cells of larvae and observed an alternation in cellular redox signaling in response to intra- or extracellular activation by oxidative stimuli. In this experiment, a large production of ROS was observed while in our previous study, we did not see any such change in the ROS level of larval gut cells treated with the MnFe_2_O_4_@poly(*t*BGE-*alt*-PA) nanocomposite.^26^ Furthermore, the climbing pattern of adult flies is used to study the anomalous geotaxis movement that results from balancing gene alteration. In this study, a climbing experiment was conducted to see the impact of the ZnFe_2_O_4_@poly(*t*BGE-*alt*-PA) nanocomposite on adult flies’ locomotive coordination. At low concentrations of the nanocomposite, adult flies displayed fewer negative geotaxis behavior, while at higher concentrations of nanocomposite, more significant changes were seen. However, in our previous study, no significant changes were seen in the climbing pattern of adult flies treated with the MnFe_2_O_4_@poly(*t*BGE-*alt*-PA) nanocomposite.^26^ Next, the body weight of adult flies varied when treated with different concentrations of the ZnFe_2_O_4_@poly(*t*BGE-*alt*-PA) nanocomposite. A significant reduction in the average body weight of adult flies was seen when compared with control. Also, morphological changes were seen in the wing venation pattern and eye color of treated flies indicating the deformities caused by nanocomposite.^50^ However, no significant changes were seen in the average body weight and phenotypic pattern of MnFe_2_O_4_@poly(*t*BGE-*alt*-PA) nanocomposite-treated adult flies.^26^

Based on the results, the ZnFe_2_O_4_@poly(*t*BGE-*alt*-PA) nanocomposite in *D. melanogaster* was found to be unsafe to use. Prior to using this nanocomposite, there is a need to perform extensive toxicological studies in other *in vivo* models. If this nanocomposite will be found nontoxic to other *in vivo* models, then it may be used in various biomedical applications.

## 5 Conclusions

In this study, the ZnFe_2_O_4_@poly(*t*BGE-*alt*-PA) nanocomposite synthesized earlier by our group was tested for its toxicological behavior in fruit flies as an *in vivo* model. This model is suitable for toxicological studies, as it has a short life cycle and a high degree of genetic homology to humans and is easy to handle in the lab. The nanocomposite was given orally to fruit flies and their larvae after mixing in food vials. During tests, an increase in ROS levels was observed, indicating high oxidative stress in gut cells leading to their cell death. The nanocomposite also showed changes in neuronal activities as well as wing venation patterns along with eye color. Since the tested nanocomposite exhibited genotoxic as well as cytotoxic effects in *D. melanogaster*, there is a need to explore its extensive toxicological aspects in a rodent model. Based on the further results, if the nanocomposite is nontoxic then only it may be used for biological applications.

## Supporting Information Available

Comet assay was performed to study DNA damage in the hemolymph of treated third instar larvae of fruit flies after oral ingestion of the ZnFe_2_O_4_@poly(*t*BGE-*alt*-PA) nanocomposite (PDF)

## Notes

This article does not contain any studies with human or animal subjects.
