# Supplementary material for: In Vivo Toxicological Analysis of the ZnFe2O4@poly(tBGE-alt-PA) Nanocomposite: A Study on Fruit Fly
Source: ACS Omega. 2024 Jan 16;9(6):6549–55. doi: 10.1021/acsomega.3c07111 (PMC10870305; doi:10.1021/acsomega.3c07111)
Supplement: Supplementary file 1 — ao3c07111_si_001.pdf [file ao3c07111_si_001.pdf]

Supplementary Information

In vivo toxicological analysis of $ZnFe_2O_4$@poly(*t*BGE-*alt*-PA) Nanocomposite: A study on Fruit Fly

Shaily Chauhan[1,2#], Seekha Naik[3#], Rohit Kumar[1,2], Janne Ruokolainen[4*], Kavindra Kumar Kesari[4,5*], Monalisa Mishra[3*], Piyush Kumar Gupta[1,2,6*]

1. Department of Life Sciences, Sharda School of Basic Sciences and Research, Sharda University, Greater Noida, 201310, Uttar Pradesh, India
2. Centre for Development of Biomaterials, Sharda University, Greater Noida, 201310, Uttar Pradesh, India
3. Department of Life Science, National Institute of Technology, Rourkela, 769008, Odisha, India
4. Department of Applied Physics, School of Science, Aalto University, Espoo, 02150, Finland
5. Research and Development Cell, Lovely Professional University, Phagwara, 144411, Punjab, India
6. Department of Biotechnology, Graphic Era (Deemed to Be University), Dehradun, 248002, Uttarakhand, India

[#]Both authors contributed equally as the first author.

***Corresponding authors:**

Dr. Kavindra Kumar Kesari
Email id. kavindra.kesari@aalto.fi

Dr. Piyush Kumar Gupta
Email id. piyush.kumar1@sharda.ac.in, dr.piyushkgupta@gmail.com

Dr. Monalisa Mishra
Email id. monalisam@nitrkl.ac.in

Prof. Janne Ruokolainen
Email: janne.ruokolainen@aalto.fi

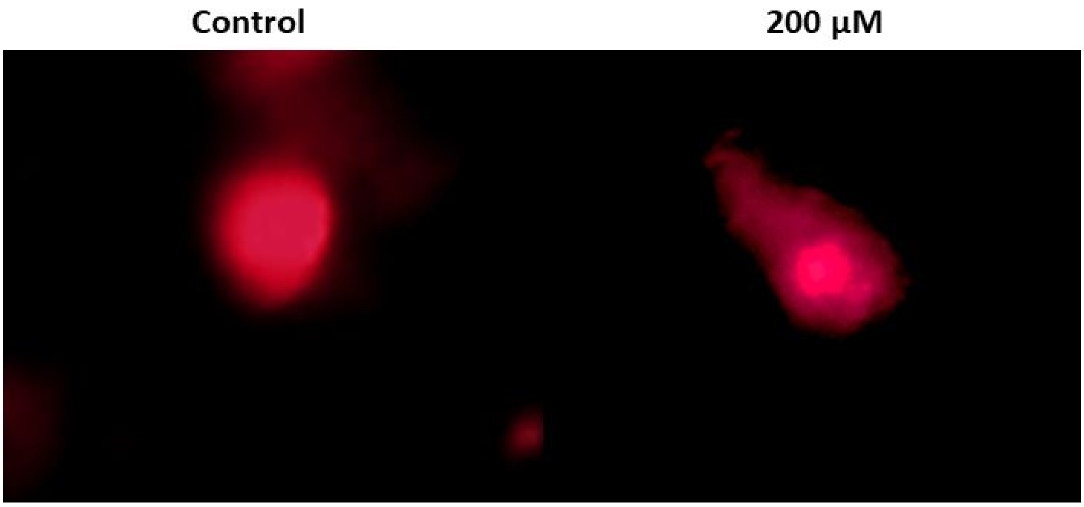

Figure S1. Comet assay to study DNA damage in the hemolymph of 3rd instar larvae after oral ingestion of $ZnFe_2O_4$@poly(*t*BGE-*alt*-PA) nanocomposite.
